# Gender differences in the provision of injection initiation assistance: a comparison of three North American settings

**DOI:** 10.1186/s12954-018-0270-6

**Published:** 2018-12-04

**Authors:** Stephanie A. Meyers, Ayden Scheim, Sonia Jain, Xiaoying Sun, M. J. Milloy, Kora DeBeck, Kanna Hayashi, Richard S. Garfein, Dan Werb

**Affiliations:** 1 School of Social Work, College of Health and Human Services, San Diego State University, 5500 Campanile Drive, San Diego, CA 92182 USA; 2 Division of Infectious Diseases and Global Public Health, Department of Medicine, University of California San Diego, 9500 Gilman Drive, La Jolla, CA 92093-0507 USA; 3 British Columbia Centre for Excellence in HIV/AIDS, 608-1081 Burrard Street, Vancouver, BC V6Z 1Y6 Canada; 4 Li Ka Shing Knowledge Institute, St. Michael’s Hospital, 30 Bond Street, Toronto, ON M5B 1T8 Canada

**Keywords:** Injection initiation, People who inject drugs, Gender, San Diego, Tijuana, Vancouver

## Abstract

**Aim:**

Individuals experience differential risks in their initiation into drug injecting based on their gender. Data suggest women are more likely to be injected after their initiator and to share injection equipment. Little is known, however, regarding how gender influences the risk that people who inject drugs (PWID) may assist others into injection initiation. We therefore sought to investigate the role of “initiator” gender in the provision of injection initiation assistance across multiple settings.

**Methods:**

We employed data from *PReventing Injecting by Modifying Existing Responses* (PRIMER), a multi-cohort study investigating factors influencing injection initiation assistance provision. Data were drawn from three cohort studies of PWID in San Diego, USA (STAHR II); Tijuana, Mexico (El Cuete IV); and Vancouver, Canada (VDUS). Site-specific logistic regression models were fit, with lifetime provision of injection initiation assistance as the outcome and gender as the independent variable.

**Results:**

Overall, 3.2% (24/746) of the women and 4.6% (63/1367) of the men reported providing injection initiation assistance. In Tijuana, men were more than twice as likely to have provided injection initiation assistance after controlling for potential confounders (adjusted odds ratio = 2.17, 95% confidence interval: 1.22–3.84). Gender was not significantly associated with providing injection initiation assistance in other sites.

**Conclusion:**

We identified that being male in Tijuana, specifically, was associated with providing injection initiation assistance, which could inform targeted outreach aimed at reducing the influence of PWID populations on non-injectors in this site. This will likely require that existing interventions address gender- and site-specific factors for effectiveness.

## Background

People who inject drugs (PWID) are disproportionately impacted by blood-borne diseases such as HIV and hepatitis C [1]. Drug injecting is also a key risk factor for overdose, particularly with the emergence of high-potency opioids such as fentanyl [2]. Relatedly, recently initiated PWID have been shown to be at particularly high risk of blood-borne disease transmission [3, 4]. This is likely due to a reliance among inexperienced PWID on more established PWID to perform injections, which leads to a concomitant increase in the risk of sharing used injecting equipment [3].

Past literature highlights the importance of gender in injection initiation processes and related risks [4–8]. Women are particularly vulnerable to the risk of blood-borne disease transmission during injection initiation events, as they are more likely to be initiated by a male intimate partner, share drug preparation equipment, and be injected after their initiator [5, 6].

Though past research has established these gender differences in the initiation process of PWID [4–8], a more fulsome understanding of the gendered processes by which individuals are initiated into drug injecting is crucial to preventing transitions into this mode of drug consumption and its corresponding harms. This study, therefore, sought to determine how gender may influence the risk that PWID provide injection initiation assistance to those who have never injected, across distinct geographic and cultural settings (i.e., San Diego, USA; Tijuana, Mexico; and Vancouver, Canada).

## Methods

### Data collection

*PReventing Injecting by Modifying Existing Responses* (PRIMER) investigates structural factors and interventions that may be effective in reducing the risk that PWID initiate others into injection. The PRIMER study methodology and rationale have been previously described in full [9]. In brief, PRIMER includes quantitative data pooled beginning in August 2014 from existing prospective community-recruited cohort studies of PWID: the *Proyecto El Cuete IV* (ECIV) cohort (Tijuana, Mexico); the *Study of Tuberculosis, AIDS, and Hepatitis C Risk* (STAHR II) cohort (San Diego, USA); the linked *Vancouver Injection Drug Users Study* (VDUS); and the *AIDS Care Cohort to evaluate Exposure to Survival Services* (ACCESS; Vancouver, Canada). All cohorts relied on convenience sampling to recruit people who use drugs, though sampling for PRIMER began at different times across sites. Additionally, VDUS and ACCESS recruited participants aged 14 years and older, whereas STAHR II and ECIV recruited those aged 18 and older. For the present study, eligibility was restricted to individuals who reported injection drug use within the 30 days prior to baseline and participants provided consent prior to enrollment. All cohort surveys collected data on sociodemographic factors and those related to drug use, including involvement in injection initiation assistance provision. All study sites received ethical approval from their local institutional review boards (IRBs) [9], and PRIMER was approved by the University of California, San Diego, IRB.

### Statistical analyses

Cross-sectional analyses were performed at the PRIMER baseline, defined as the visit when injection drug use initiation questions were first introduced to each cohort. We defined the outcome as ever having provided injection initiation assistance (yes vs. no). The primary independent variable was participant gender (i.e., males vs. females; only 5 participants [< 0.1%] self-identified as transgender, and we were unable to independently assess this group). In line with previous studies, and due to shared vulnerabilities between the two groups, transgender participants were considered within the female group [10]. We also assessed the following covariates across all three sites: age, years since first injection, housing status, and marital status. Data on self-reported lifetime non-injection and injection use of methamphetamine, cocaine, and heroin were available for participants in San Diego and Tijuana and were included as potential covariates for analyses specific to these sites. All analyses were undertaken separately by study site (i.e., San Diego, Tijuana, and Vancouver). Missing cases comprised less than 5% of the sample (*n =* 35) and were excluded from the analyses [11].

As determined a priori, variables associated with ever providing injection initiation assistance in bivariate analysis at the *p* < 0.05 level were retained for inclusion in the multivariable model; participant age and years since first injection were also included regardless of bivariate significance. We then employed a multivariable logistic regression modeling approach for each cohort in which all variables of interest were entered simultaneously. Each final multivariable model included the primary variable of interest (gender), age, years since first injection, and any non-injection or injection drug use variables that retained significance. All analyses were conducted using SAS On Demand for Academics (SAS Institute Inc., Cary, North Carolina, USA).

## Results

Participant baseline characteristics are presented in Table 1. Table 2 presents site-specific bivariate and multivariable results. Of the 746 women sampled, 24 (3.2%) reported providing injection initiation assistance. For the 1367 men recruited, 63 (4.6%) reported providing injection initiation assistance. In Tijuana, being a man was associated with ever having provided injection initiation assistance (adjusted odds ratio [AOR] = 2.17, 95% confidence interval [CI]: 1.22, 3.84, *p* = 0.01). In both Vancouver and San Diego, gender was not significantly associated with provision of injection initiation assistance. In Vancouver, injection initiation assistance was associated with years since first injection (AOR = 1.04, 95% CI: 1.02, 1.06, *p* < 0.01) and inversely associated with age (AOR = 0.95, 95% CI: 0.93, 0.97, *p* < 0.01). In San Diego, injection initiation assistance provision was inversely associated with age (AOR = 0.95, 95% CI: 0.92, 0.98, *p* < 0.01). None of the non-injection or injection drug use variables were significantly associated with providing injection initiation assistance in the multivariable models.

**Table 1:** Injection initiation assistance provision and related factors among people who inject drugs in San Diego, USA; Tijuana, Mexico; and Vancouver, Canada (*n* = 2113)

Categorical variables	San Diego (*N* = 347) *n* (%)	Tijuana (*N* = 532) *n* (%)	Vancouver (*N* = 1234) *n* (%)
*Helped someone initiate injection (lifetime)*			
Yes	130 (37.5)	76 (14.3)	288 (23.3)
No	217 (62.5)	456 (85.7)	946 (76.7)
*Gender*			
Men	249 (71.8)	327 (61.5)	791 (64.1)
Women	98 (28.2)	205 (38.5)	443 (35.9)
*Housing status*			
Stable housing	179 (51.6)	330 (62.0)	389 (31.5)
Other	168 (48.4)	202 (38.0)	845 (68.5)
*Marital status*			
Married	41 (11.8)	245 (46.1)	171 (13.9)
Other	306 (88.2)	287 (54.0)	1063 (86.1)
*Non-injected heroin (lifetime)*			
Yes	263 (75.9)	289 (54.3)	–
No	84 (24.2)	243 (45.7)	–
*Non-injected cocaine (lifetime)*			
Yes	317 (91.4)	361 (67.9)	–
No	30 (8.7)	171 (32.1)	–
*Non-injected methamphetamine (lifetime)*			
Yes	328 (94.5)	468 (88.0)	–
No	19 (5.48)	64 (12.03)	–
*Injected heroin (lifetime)*			
Yes	298 (85.9)	531 (99.8)	–
No	49 (14.1)	1 (0.2)	–
*Injected cocaine (lifetime)*			
Yes	259 (74.6)	321 (60.3)	–
No	88 (25.4)	211 (39.7)	–
*Injected methamphetamine (lifetime)*			
Yes	309 (89.05)	471 (88.53)	–
No	38 (10.95)	61 (11.47)	–
*Age (mean years)*	46.84 (SD 11.28)	41.05 (SD 8.68)	45.76 (SD 11.62)
*Years since first injection (mean years)*	23.91 (SD 13.12)	19.90 (SD 9.46)	23.98 (SD 12.86)

Note: *SD* standard deviation

**Table 2:** Bivariate and multivariable associations with injection initiation assistance among people who inject drugs in San Diego, USA; Tijuana, Mexico; and Vancouver, Canada

	San Diego (*n* = 347)		Tijuana (*n* = 532)		Vancouver (*n* = 1234)	
	Unadjusted OR (95% CI)	Adjusted OR (95% CI)	Unadjusted OR (95% CI)	Adjusted OR (95% CI)	Unadjusted OR (95% CI)	Adjusted OR (95% CI)
Age	0.97 (0.95–0.99)**	0.95 (0.92–0.98)**	1.00 (0.97–1.02)	0.97 (0.92–1.01)	0.98 (0.97–0.99)**	0.95 (0.93–0.97)***
Years since first injection	0.99 (0.97–1.01)	1.03 (0.99–1.06)	1.01 (0.99–1.04)	1.03 (0.98–1.07)	1.00 (0.99–1.01)	1.04 (1.02–1.06)***
Gender (ref = women)	1.18 (0.73–1.92)	1.29 (0.78–2.14)	2.07 (1.19–3.59)**	2.17 (1.22–3.84)*	0.99 (0.75–1.30)	1.10 (0.83–1.46)
Marital status (ref = other)	0.75 (0.37–1.51)	–	0.78 (0.48–1.28)	–	1.43 (1.00–2.05)	–
Housing status (ref = other)	0.67 (0.43–1.04)	–	1.06 (0.64–1.75)	–	1.21 (0.92–1.60)	–
Non-injected heroin use (ref = no)	1.03 (0.62–1.72)	–	1.63 (0.99–2.71)	–	–	–
Non-injected cocaine use (ref = no)	1.72 (0.74–3.99)	–	1.63 (0.93–2.86)	–	–	–
Non-injected methamphetamine use (ref = no)	1.72 (0.61–4.90)	–	1.19 (0.54–2.61)	–	–	–
Injected heroin use (ref = no)	1.04 (0.55–1.94)	–	–	–	–	–
Injected cocaine use (ref = no)	1.07 (0.65–1.76)	–	1.31 (0.79–2.19)	–	–	–
Injected methamphetamine use (ref = no)	2.07 (0.95–4.53)	–	3.55 (1.08–11.62)*	3.17 (0.96–10.52)	–	–

Note: *OR* odds ratio, *CI* confidence interval

* *p* < .05, ** *p* < .01, ***p < .001

## Discussion

Ever providing injection initiation assistance was associated with being male in Tijuana, but not in San Diego or Vancouver. Age was inversely associated with this behavior in both San Diego and Vancouver, and a higher number of years since first injection was associated with this behavior in Vancouver. These findings illuminate the differing role of gender in injection initiation across sites and have implications for efforts to prevent injection drug use and related harms.

Previous research has highlighted the impact of gender on injection-related risks and has reported on gender-specific pathways to injection initiation [4–8]. Findings from the current study suggest that gender may, to some extent, determine the risk that PWID provide injection initiation assistance. Further, this appears to be highly context-specific and likely related to the particular social norms and policy practices that shape local injecting practices. In Tijuana, arbitrary policing practices encourage secrecy on behalf of PWID and foster an environment where individuals are more likely to inject alone to avoid harassment by law enforcement [12]. This may account for the lower prevalence of injection initiation assistance we observed in Tijuana. Women in Tijuana are more likely to inject in their homes and with trusted individuals [13], which may make them less likely to inject in the presence of injection-naïve individuals, or at venues in which initiation commonly occurs (i.e., shooting galleries) [14]. These gender-specific patterns are likely less entrenched in San Diego and Vancouver, potentially as a result of less intense risks for physical danger arising from law enforcement practices or street violence [15]. Future qualitative research is needed to fully investigate this hypothesis.

We also note the contrasting risk for injection initiation assistance associated with age and years injecting among participants in Vancouver. This implies that younger participants who began injecting early were more likely to have provided injection initiation assistance compared with older individuals who have been injecting for the same number of years. Efforts to disrupt the process of injection initiation may be most effective in Vancouver if focused on younger individuals with more experience injecting drugs.

### Limitations

This study has limitations typical of observational cross-sectional research. Non-probability sampling was used for participant recruitment, and we cannot assume generalizability for populations of PWID in each study setting [11]. Secondly, we relied on self-report, and underreporting of experiences of initiating others into injecting is likely given that it is highly stigmatized [16]. Additionally, it is possible that providing injection initiation assistance is differentially under-reported both by gender and across sites due to existing gender norms and stigma across the sites investigated.

### Implications

To our knowledge, this is the first study investigating the role of gender in assisting others to initiate injecting across multiple countries. The present study indicates that the likelihood of initiating others into injection drug use is impacted by one’s gender in Tijuana, one’s age in San Diego and Vancouver, and the number of years since first injection in Vancouver. These findings can provide foundations for efforts to prevent injection initiation across sites as well as among specific high-risk subpopulations. We note that these findings have implications for interventions seeking to prevent PWID facilitating the entry of others into injecting. Specifically, pathways to initiating others appear to be highly gendered and distinct across local contexts. As such, preventing the transition of individuals into injection drug use will likely require that existing interventions (such as Change the Cycle [17]) adapt to address site- and population-specific gender dynamics to ensure effectiveness. Future injection prevention efforts should focus on providing gender- and context-specific prevention programs, like one-to-one social learning programs [17, 18], targeting men who inject drugs in Tijuana and young PWID in San Diego and Vancouver.

## Abbreviations

ACCESS: AIDS Care Cohort to evaluate Exposure to Survival Services study
ECIV: Proyecto El Cuete IV study
HCV: Hepatitis C virus
HIV: Human immunodeficiency virus
PRIMER: PReventing Injecting by Modifying Existing Responses study
PWID: People who inject drugs
STAHR II: Studying Tuberculosis AIDS and Hepatitis C Risk study
VDUS: Vancouver Injection Drug Users Study
